# Primary Invasive Vaginal Carcinoma Associated with Complete Utero-Vaginal Prolapse: A Case Report and Literature Review

**DOI:** 10.3390/jcm14134622

**Published:** 2025-06-30

**Authors:** Maciej Korniluk, Weronika Niemyjska-Dmoch, Beata Gil-Sobocińska, Joanna Kabat, Lubomir Bodnar, Grzegorz Szewczyk

**Affiliations:** 1 Department of Obstetrics, Gynecology and Gynecological Oncology, St. John Paul 2nd Mazovia Regional Hospital in Siedlce, 08-110 Siedlce, Poland; weronikaniemyjska@gmail.com (W.N.-D.); gszewczyk@szpital.siedlce.pl (G.S.); 2 Department of Obstetrics, Perinatology and Gynecology, Medical University of Warsaw, 02-091 Warszawa, Poland; 3 Department of Clinical Oncology and Radiotherapy, St. John Paul 2nd Mazovia Regional Hospital in Siedlce, 08-110 Siedlce, Poland; bsobocinska@szpital.siedlce.pl (B.G.-S.); lbodnar@szpital.siedlce.pl (L.B.); 4 Department of Radiology, St. John Paul 2nd Mazovia Regional Hospital in Siedlce, 08-110 Siedlce, Poland; jkabat1@szpital.siedlce.pl; 5 Faculty of Medical and Health Sciences, University of Siedlce, 08-110 Siedlce, Poland; 6 1st Department of Obstetrics and Gynecology, Centre of Postgraduate Medical Education, 02-097 Warsaw, Poland; 7 Department of Obstetrics and Gynecology, St. Sophia Hospital, 01-004 Warsaw, Poland

**Keywords:** vaginal carcinoma, complete utero-vaginal prolapse, surgical treatment, prolapse reconstruction, radiotherapy

## Abstract

**Background:** Primary vaginal cancer is a rare gynecological condition. We present a case of complete utero-vaginal prolapse complicated by primary invasive vaginal carcinoma. To our knowledge, only a few similar cases have been reported in our region. **Case Report:** A 77-year-old woman, gravida two and para two, was admitted for treatment of pelvic organ prolapse. The patient reported an eight-year history of uterine bulging but had not used a pessary. The gynecological examination revealed a complete manually irreducible utero-vaginal prolapse with an ulcerative lesion on the right posterolateral vaginal wall. The histological examination diagnosed an HPV-independent keratinizing squamous cell carcinoma, grade I. Comprehensive imaging showed no evidence of metastasis. The patient underwent radical hysterectomy, bilateral adnexectomy, complete resection of the vaginal mass, and pelvic lymphadenectomy. The histopathological examination confirmed clear surgical margins. According to the International Federation of Gynecology and Obstetrics (FIGO) staging system, the disease was classified as stage I vaginal cancer. Postoperatively, the patient received radiotherapy (45 Gy) and high-dose-rate brachytherapy (14 Gy). **Conclusions:** The co-occurrence of vaginal cancer and utero-vaginal prolapse is exceedingly rare. Surgical intervention followed by radiotherapy is the most common treatment approach. Given the aggressive nature of the disease, comprehensive follow-up is essential. Further research is needed to determine whether long-term genital prolapse increases the risk of vaginal carcinoma.

## 1. Introduction

Primary vaginal cancers are among the rarest gynecological malignancies [1]. These tumors account for 1–3% of all gynecological neoplasms and are most frequently diagnosed in women aged 60 to 70 years [2,3]. According to the Global Cancer Observatory, there were 18,800 cases of vaginal cancer worldwide in 2022, accounting for 0.1% of all diagnosed cancers and 0.4% of all female cancers [4]. The coexistence of uterine prolapse and vaginal cancer is an even rarer occurrence [5].

Typical manifestations of this condition include vaginal bulging, bleeding, and the presence of a mass in the upper vagina. Other symptoms may include pelvic pain, dyspareunia, or urinary symptoms such as dysuria or urgency, particularly if the tumor invades adjacent structures. The nonspecific nature of these symptoms often leads to delayed diagnosis, particularly in early-stage disease.

Approximately 90% of primary vaginal cancers are squamous cell carcinomas, making it the most common histological subtype. Several risk factors have been identified, including chronic human papillomavirus (HPV) infection, chronic irritation and inflammation, smoking, sexually transmitted diseases (STDs), diethylstilbestrol exposure during pregnancy, and pessary therapy [6,7,8,9,10,11,12,13].

Diagnosis of vaginal cancer typically involves a gynecological examination, colposcopy, and biopsy of suspicious lesions. Imaging modalities such as magnetic resonance imaging (MRI) or positron emission tomography–computed tomography (PET-CT) are used to assess the tumor extent, lymph node involvement, and distant metastases. Early detection is critical, as advanced-stage disease is associated with poorer outcomes.

Treatment is multidisciplinary and often challenging. Surgical management is the primary approach for early-stage disease, particularly when located in the upper vagina. In more advanced cases, radiotherapy or chemoradiation may be more appropriate.

The prognosis for vaginal cancer varies significantly based on stage at diagnosis. Factors associated with improved outcomes include early-stage diagnosis, smaller tumor size, absence of lymph node involvement, and HPV status. The rarity of vaginal cancer and its association with uterine prolapse additional challenges, as prolapse may delay diagnosis or complicate surgical planning.

The scarcity of primary vaginal cancer cases hinders the conduct of large-scale clinical trials, complicating the development of evidence-based treatment guidelines.

## 2. Materials and Methods

A systematic search of the literature indexed on PubMed was conducted using EndNote (https://web.endnote.com) (Clarivate) with a combination of keywords and text words, including “pelvic organ prolapse,” “genital prolapse,” “uterus prolapse,” and “uterine prolapse,” with coexisting “vaginal cancer,” “vaginal carcinoma,” and “vaginal neoplasm.” A complete search strategy is provided in the Supplementary Material S1. In accordance with the PRISMA statement [14], the study objectives, eligibility criteria, search strategy, and data inclusion process were rigorously defined and followed. The quality assessment, data extraction process, and potential biases have been thoroughly described (Supplementary Material S2). Only articles written in English were included in the analysis. The review included case reports published between 1945 and 2024.

To identify additional relevant articles, a manual search was conducted using the reference lists of key articles. The records retrieved from database searches were merged, and duplicates were removed using EndNote (Clarivate). Case reports and records meeting the inclusion criteria were included in the review process and are summarized in Table 1.

## 3. Case Report

### 3.1. Symptoms and Diagnostics

**Figure 1:**
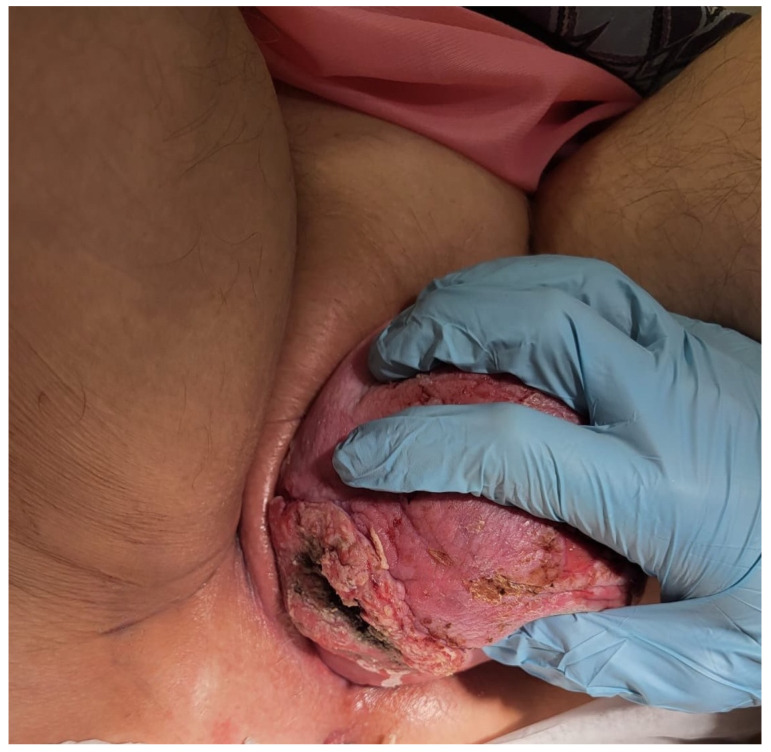
Ulcerative tissue formation in the right posterolateral wall detected during initial visit.

**Figure 2:**
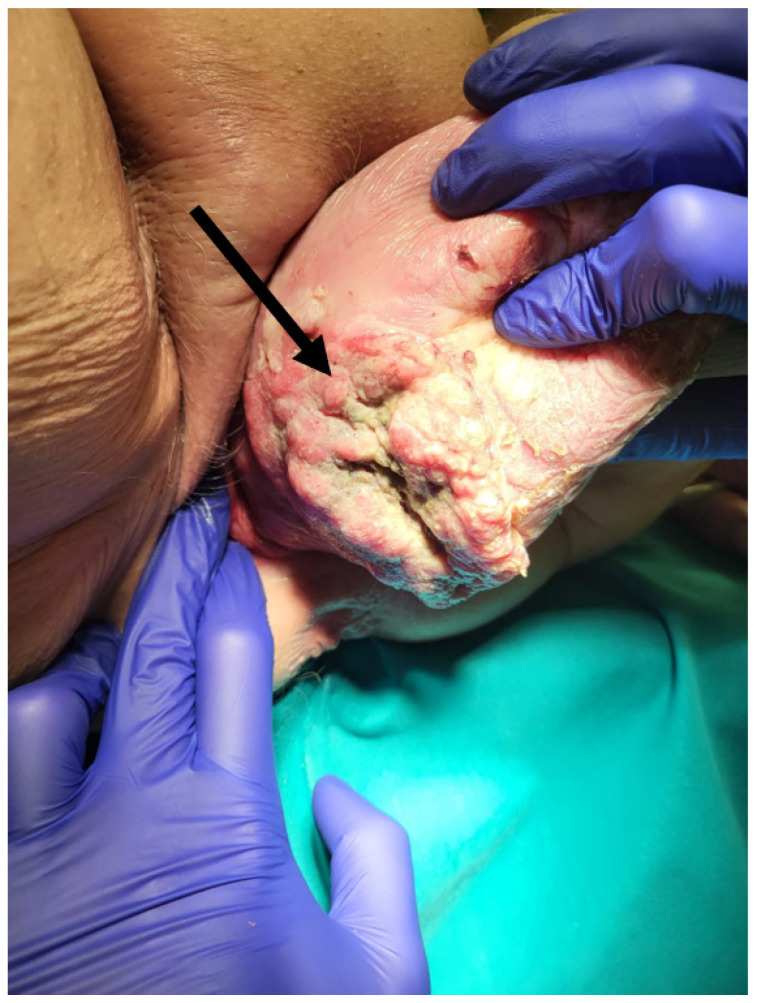
Irreducible uterovaginal prolapse with vaginal carcinoma (arrow).

**Figure 3:**
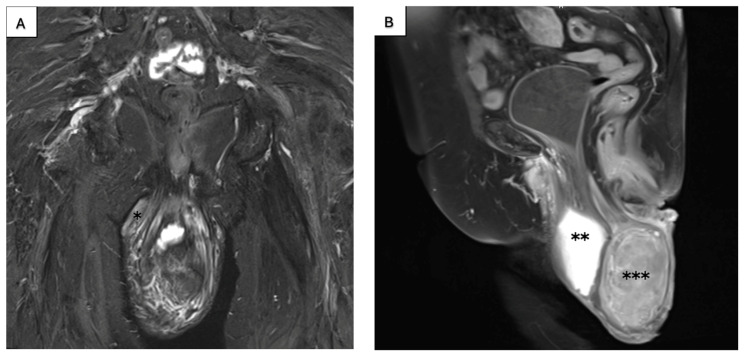
(**A**) T2-weighted magnetic resonance in coronal plane. (**B**) T1-weighted magnetic resonance in sagittal plane. Images showing a stage IV completely prolapsed uterus with mass. * vaginal carcinoma, ** bladder, *** prolapsed uterus.

A 77-year-old multiparous woman was referred to the hospital with an eight-year history of uterovaginal prolapse. The patient reported no additional symptoms. Her medical history included hypertension and type II diabetes. The gynecological examination revealed a stage 4 uterovaginal prolapse, irreducible by manual repositioning, and an ulcerative tissue formation (5 × 8 cm) on the right posterolateral vaginal wall (Figure 1). According to the International Classification of Prolapse of the Reproductive Organ (POPQ), the prolapse was classified as POPQ IV [37]. The vaginal atrium margins measured up to 4 cm in length (Figure 2). Macroscopically, the cervix appeared normal; during the gynecological examination, the adnexa were non-palpable, and the parametria were free of lesions. The histological analysis of the mass revealed an HPV-independent keratinizing squamous cell carcinoma, grade 1, with p53 positivity and p16 negativity. MRI and CT imaging revealed a uterus measuring 88 × 45 mm with a heterogenous endometrium of 10 mm thickness. An irregular lesion was identified on the right posterior vaginal wall, measuring 56 mm in length, 18 mm in thickness, and 67 mm in width, without clear evidence of infiltration into adjacent structures (Figure 3). The ovaries were not visualized. The retroperitoneal, mesenteric, and pelvic lymph nodes were not enlarged. No free fluid was observed in the abdominal cavity. The urinary bladder exhibited smooth wall contours, and the rectum appeared normal. No evidence of distant organ metastasis was identified. The patient was clinically and radiologically staged as FIGO stage I and, based on the multidisciplinary team’s recommendation, deemed eligible for primary surgery. The scope of the procedure included a radical abdominal hysterectomy with vaginal resection encompassing the tumor mass, bilateral adnexectomy, and pelvic lymph node removal.

### 3.2. Treatment

After obtaining informed consent, the procedure was performed. Intraoperatively, no free fluid was observed, and the abdominal organs and adnexa appeared intact on gross examination. The prolapse was successfully reduced under general anesthesia by applying precise external pressure while simultaneously pulling the uterus through the pelvis. The uterus was slightly enlarged. The parametrium and vagina below the tumor tissue were dissected while maintaining oncological sterility. A hysterectomy with adnexectomy and vaginectomy was performed, followed by bilateral pelvic lymphadenectomy performed via midline laparotomy. The greatest challenge during the procedure was the low vaginectomy and the dissection of the urinary bladder from the free margin boundary.

**Figure 4:**
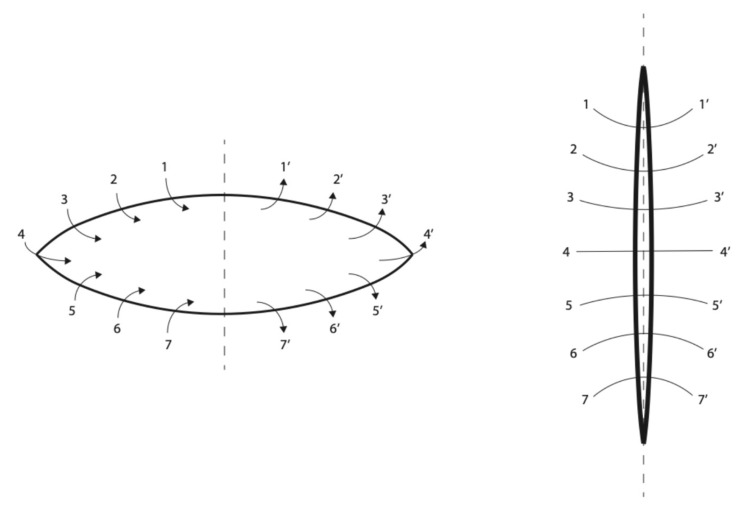
The vaginal vault was sutured with a continuous suture starting at 11 o’clock from the left to the right side. Arrows indicate direction of surgical suture. Arabic numbers indicate the order of surgical punctures. **Left side**—condition before surgical treatment. **Right side**—condition after surgical supply.

The vaginal vault was closed with a continuous absorbable suture, starting at the 11 o’clock position (Figure 4). The fascia was reconstructed to strengthen the surrounding tissues [38,39,40,41]. Sacrovaginopexy was not performed. The postoperative period was uneventful, with no complications reported.

### 3.3. Histology Postoperative Report

**Figure 5:**
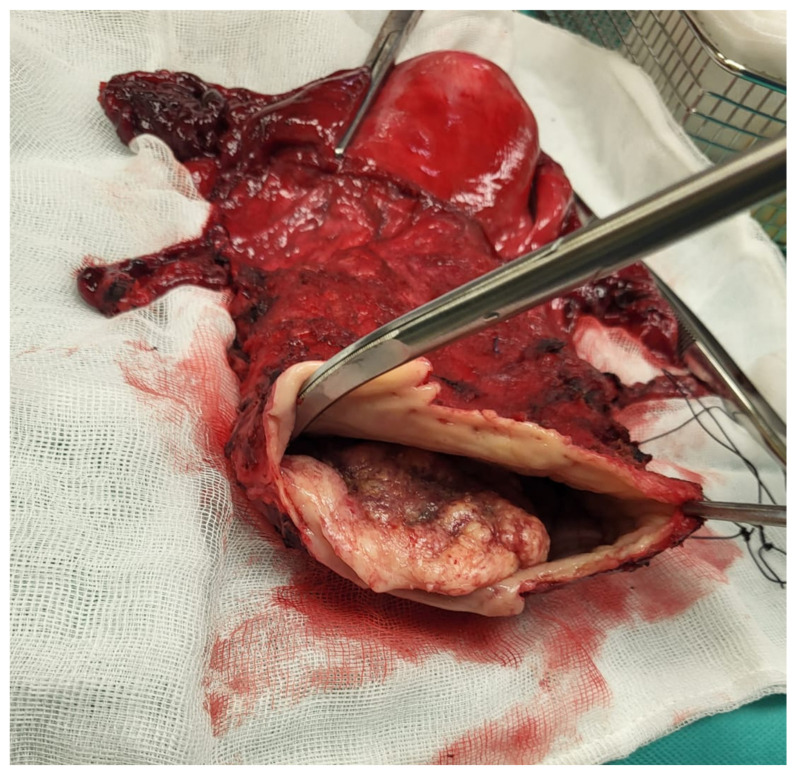
Resected specimen showing uterus with Fallopian tubes and ovaries, cervix, and parametrium with anterior and posterior walls of vagina with mass. Resection margins are clearly visible.

The pathological examination of the excised materials confirmed an HPV-independent keratinizing squamous cell carcinoma, grade 2, with p53 positivity, p16 unspecific, and a high Ki67 proliferation index. The deepest infiltration measured 11 mm. The lymphovascular space involvement was positive, and no neuroinvasion was observed. The deep tissue margin was 1,5 mm. The surgical margins at the 9, 12, and 3 o’clock positions were free of tumor infiltration, with the 6 o’clock position margin measuring 10 mm. A leiomyoma was identified in the uterine corpus. The endometrium exhibited cystic endometrial atrophy. The cervix was covered with multilayered squamous epithelium showing keratinization, without dysplasia. Bilateral parametrial structures showed no focal lesions. The right adnexa were free of focal lesions. The left fallopian tube contained a simple cyst. The left ovary showed no focal lesions. The TNM Classification of Malignant Tumors (TNM) was pT1b N0 R0 (pTNM according to AJCC 8th edition) [42]. All excised lymph nodes were tumor-free (Figure 5). According to the International Federation of Gynecology and Obstetrics (FIGO) staging system, the disease was classified as stage I vaginal cancer [43].

### 3.4. Adjuvant Treatment and Follow-Up

Postoperatively, the patient underwent radiation therapy to the affected area, including the lodge area, vagina, and regional pelvic lymph nodes, with a total dose of 45 Gy. High-dose-rate brachytherapy (BRT HDR) was also administered, with a total dose of 14 Gy. The patient tolerated the treatment well, with no reported adverse effects.

**Figure 6:**
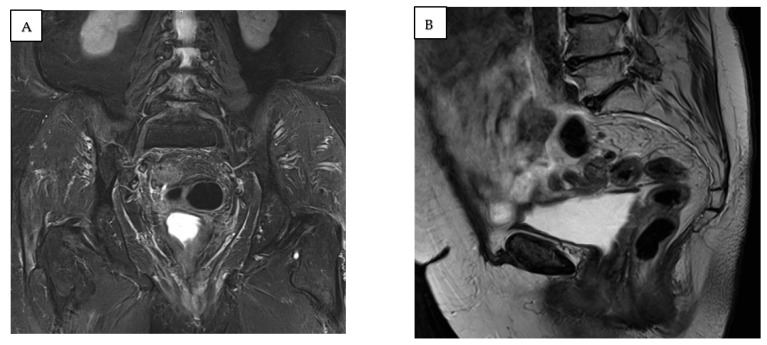
(**A**) T2-weighted magnetic resonance in coronal plane. (**B**) T2-weighted magnetic resonance in sagittal plane. Images performed eight months after surgery. No evidence of the disease recurrence was observed.

**Figure 7:**
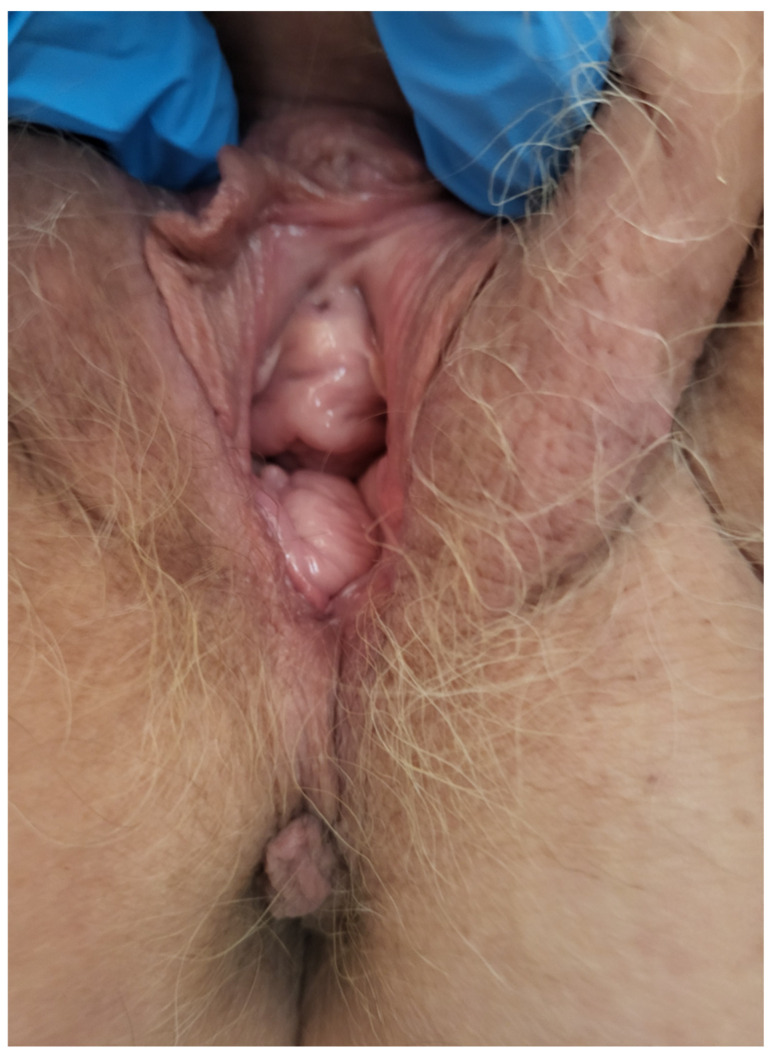
The appearance of the patient’s vulva after 12 months of observation.

Eight months after surgery, magnetic resonance imaging showed no evidence of disease recurrence or enlarged lymph nodes (Figure 6). At the 12-month follow-up, the patient’s condition remained satisfactory, with no signs of vaginal prolapse, urinary incontinence, or disease recurrence (Figure 7).

## 4. Results

### 4.1. Study Assessment

**Figure 8:**
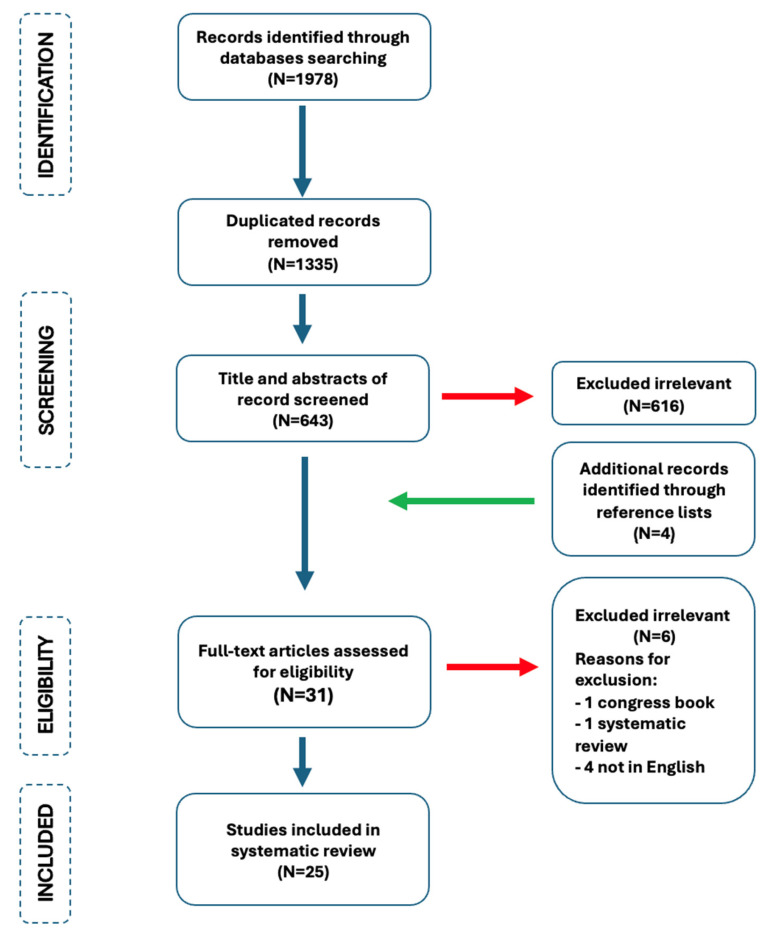
PRISMA 2020 research process.

A systematic search of electronic databases, conducted in accordance with PRISMA guidelines, yielded 1978 results. The inclusion criteria for the study were as follows: full-text case reports; studies describing patients with pelvic organ prolapse and concurrent vaginal cancer; studies published between 1945 and 2024; articles written in English. After removing 1335 duplicates, 643 records were screened. Of these, 616 were excluded based on the following criteria: studies focusing solely on vaginal cancer without concurrent pelvic organ prolapse; another gynecological cancer associated with pelvic organ prolapse; studies with unclear reporting of results; non-case report study design. Additionally, four records were included following a manual search of the reference lists of key articles. Thirty-one full-text articles were assessed for eligibility. Six irrelevant cases were excluded due to lack of relevance to the study objectives. Following the screening process, 25 studies were considered eligible for inclusion in the review. One systematic review was excluded from further consideration to maintain the focus on primary case reports [5] (Figure 8).

### 4.2. Main Findings

A comparative analysis of 33 reported cases revealed that the patients ranged in age from 44 to 98 years, with most being postmenopausal women. The most common symptoms were vaginal bleeding and discharge (17 cases, 52%), followed by prolapse and urinary incontinence (11 cases, 33%). Detailed symptom information was unavailable for seven cases (21%). According to the International Classification of Prolapse of the Reproductive Organ (POPQ), most cases were classified as POPQ III (21 patients, 64%), while nine cases (27%) were classified as POPQ IV (total prolapse), and one case (3%) as POPQ II. Two cases (6%) did not specify the degree of prolapse.

Approximately 90% of cases were squamous cell carcinomas (N = 30, 91%) [15,17,18,20,21,22,23,24,25,26,27,29,31,32,33,34,35,36]. To our knowledge, only two reported cases (6%) included HPV status determination, both of which were negative. Unfortunately, HPV status is often unreported in published cases.

FIGO (International Federation of Gynecology and Obstetrics) staging data were available for 17 patients (52%), with the majority (eight patients, 24%) classified as FIGO I.

Treatment was multidisciplinary, with surgery as the first-line approach in 14 cases (43%), followed by radiotherapy in 10 cases (30%). In other cases, surgical treatment was discontinued due to the severity of the oncological disease, or there was insufficient information on why surgical treatment was not performed. External beam radiotherapy was the primary treatment in nine cases (27%), while chemotherapy was administered as the first-line treatment in three cases (9%). Three patients (9%) declined treatment, and one (3%) was referred for palliative care. No treatment was administered in three cases (9%).

Follow-up data were available for 22 patients (67%), with no evidence of oncological disease in nine cases (27%) after 12 months. The majority of authors did not report the effectiveness of treatment for pelvic organ prolapse disorder. Out of all the cases that were examined, 11 patients (33%) died. Seven of these (21%) died from cancer, three patients (9%) died from other reasons, and in one case (3%) the cause of death remained unknown. For the documented cases, the survival time of the patients survived ranged from eight days to 93 months.

## 5. Discussion

The co-occurrence of vaginal cancer and pelvic organ prolapse is an exceedingly rare phenomenon, resulting in limited studies. Owing to limitations in the available databases, identifying the risk factors remains challenging. The systematic review relied on isolated clinical cases, which often lack comprehensive data. However, analysis of the available literature suggests potential causative factors, including HPV infection, particularly high-risk subtypes (e.g., HPV-16, HPV-18) [44].

HPV plays a pivotal role in the etiology of vaginal squamous cell carcinoma, particularly in conjunction with cofactors such as tobacco smoking and high-risk sexual behaviors (e.g., multiple partners, early sexual debut). Preventive strategies, such as HPV vaccination and health education that promotes smoking cessation and safe sexual practices, may reduce the incidence of this rare malignancy. Notably, early-stage HPV-positive vaginal carcinomas have a more favorable prognosis than HPV-negative tumors [45]. HPV-positive status is a favorable prognostic biomarker for vaginal cancer, which is significant for risk stratification and treatment planning. Patients with HPV-negative vaginal cancer require more intensive monitoring due to a higher risk of recurrence and poorer prognosis. Further studies are needed to confirm these findings in larger populations and to investigate the molecular mechanisms responsible for the prognostic differences between HPV-positive and HPV-negative cancers.

Moreover, in the case under discussion, after biopsy, the immunohistochemical staining revealed p53 expression and p16 negativity in the tumor and adjacent vaginal mucosa. Subsequent staining of the excised material revealed p16 as unspecific. Expression of p16 is a favorable prognostic biomarker in vaginal cancer, associated with improved survival and longer disease-free intervals, primarily due to its association with HPV [46]. Overexpression of p53, observed in HPV-negative tumors, indicates a poorer prognosis and potential HPV-independent carcinogenic mechanisms, such as genetic mutations or chronic inflammation [47]. These findings highlight the importance and necessity of HPV mapping and p53/p16 immunostaining when vaginal neoplastic changes are suspected.

Unfortunately, HPV mapping and immunostaining were not performed in most of the analyzed studies, limiting a comprehensive risk factor assessment. Compiling a larger literature database could facilitate the development of a standardized management pathway for vaginal cancer.

Carcinogenic mechanisms not associated with HPV have also been demonstrated. Long-term pelvic organ prolapse, which causes chronic irritation of the vaginal epithelium, may be a potential risk factor in cases unrelated to HPV. Chronic mechanical irritation, resulting from exposure of the vaginal epithelium to friction, trauma, or localized inflammation, can lead to hyperplasia, dysplasia, and ultimately neoplastic transformation [6,8,9,25,30,32]. Irritation of the vaginal epithelium does not necessarily result from uterine prolapse. The authors reported cases associated with long-term pessary use and the risk of vaginal cancer development [7,10,12,13]. Obviously further research is needed to establish a definitive link.

In the analyzed cases, the mean age of patients with pelvic organ prolapse and concurrent vaginal cancer (approximately 74 years) was slightly higher than that of patients with vaginal cancer alone, as compared to epidemiological data reported in the literature. In most cases, the symptoms prompting patients to seek medical evaluation was remarkably similar across cases. Vaginal cancers were frequently detected incidentally during gynecological examinations. The majority of vaginal cancers are located in the upper vagina. Given the overlap of symptoms, it is prudent to maintain a high index of suspicion for vaginal cancer in patients presenting with POP, urinary tract issues (e.g., urgency, incontinence), and concomitant vaginal bleeding or discharge. In such cases, vaginal cancer should be considered. A thorough gynecological examination, complemented by targeted biopsy of any suspicious lesions suggestive of neoplasia, is fully warranted. The primary objective of these diagnostic measures is to facilitate the early-stage detection of vaginal cancer, which significantly improves the treatment outcomes and prognosis.

Each patient’s case must be evaluated on an individual basis to ensure optimal outcomes in the management of vaginal cancer co-occurring with pelvic organ prolapse (POP). Treatment planning should be preceded by comprehensive imaging studies, such as pelvic magnetic resonance imaging (MRI), computed tomography (CT), or positron emission tomography (PET-CT) to assess the tumor extent, lymph node involvement, and anatomical defects associated with POP. The primary goal is radical treatment addressing both the oncological condition and the correction of POP, while minimizing complications such as urinary symptoms. The analysis of the reported cases revealed a heterogeneity in treatment approaches, reflecting the rarity of this dual pathology and the lack of standardized guidelines. Evaluating the effectiveness of applied methods, whether surgical, radiotherapeutic, or chemotherapeutic, remains challenging. Surgery emerged as the most frequently chosen first-line treatment in the reviewed cases. In a comparative analysis of FIGO stage I, as observed in the described case, surgical treatment was not the first-line approach in only two instances. The specific surgical technique employed is beyond the scope of this study, as the outcomes depend on the tumor size, location, and surgeon expertise.

However, appropriate reconstruction of the uterine prolapse defect is a critical component of treatment, aiming to restore pelvic anatomy and function while minimizing the recurrence risk.

Reconstruction using native tissue is generally preferred over synthetic mesh in the context of vaginal cancer, despite the latter’s superior anatomical correction of POP. Synthetic meshes, such as polypropylene, provide durable support and lower POP recurrence rates, but they are associated with significant complications, including mesh erosion, infection, and chronic pain. Native tissue repair, utilizing autologous fascia or ligaments is safe, with lower complication rates, but it is less effective in preventing long-term POP recurrence. Given the coexistence of an oncological condition, native tissue repair is advocated to avoid chronic epithelial irritation, which may contribute to carcinogenesis and cancer relapse, particularly in HPV-negative tumors. This recommendation aligns with emerging evidence linking chronic irritation from foreign bodies (e.g., pessaries, meshes) to HPV-independent vaginal squamous cell carcinoma [48,49,50]. The suturing technique applied by us, which additionally reinforces surrounding tissues to prevent recurrent pelvic organ prolapse, merits further observation and analysis.

Early stages (FIGO I-II) have a better prognosis and can be treated surgically or with radiotherapy, achieving cure rates of 70–90%. In advanced stages (FIGO III-IV), the standard treatment is combined radiotherapy and chemotherapy, with 5-year survival rates of approximately 30–50%. Brachytherapy plays a critical role in delivering high radiation doses to the tumor while minimizing damage to healthy tissue. Management of vaginal cancer requires an individualized approach, considering patient preferences, the disease stage, and prognostic factors, including HPV status [45], p16 expression, p53 mutation status [46,47], tumor size, FIGO stage, lymph node involvement, and response to primary treatment [51].

Radiotherapy, particularly brachytherapy, is the cornerstone of treatment for most vaginal cancer cases, especially in advanced stages, with the addition of chemotherapy enhancing outcomes. Early-stage disease can be treated surgically or with radiotherapy, offering a better prognosis. Preventive strategies, such as HPV vaccination and safe sexual practices, may reduce the incidence of this malignancy. Furthermore, many authors emphasize the critical role of MRI imaging in treatment planning and monitoring the response.

Recurrence of vaginal cancer remains a formidable clinical challenge, with significant implications for patient prognosis and quality of life. The time to recurrence varies widely, influenced by histological subtype, HPV status, FIGO stage, and treatment modality. The literature data suggest that the median time to recurrence ranges from 6 to 18 months, with a shorter interval observed in HPV-negative tumors. Chronic epithelial irritation, such as that associated with pelvic organ prolapse (POP) or synthetic meshes used in POP repair, may elevate the recurrence risk by inducing persistent inflammation and DNA damage, potentially promoting HPV-independent carcinogenesis. Regular follow-up examinations, including imaging studies (e.g., MRI, PET-CT), in the first months post-treatment are critical for the early detection of recurrence, which may enhance salvage treatment outcomes. The literature on vaginal cancer recurrence is constrained by the malignancy’s rarity, with most data sourced from retrospective case series or studies combining vaginal and vulvar cancers, complicating the isolation of vaginal cancer-specific recurrence patterns [52,53,54].

In the present case, surgical intervention was the primary treatment. Over 12 months of follow-up, no instances of disease recurrence, vaginal cuff dehiscence, infection, or bleeding were observed following the application of our suturing technique.

In summary, co-occurring pelvic organ prolapse (POP) and vaginal cancer present a complex clinical entity characterized by significant diagnostic, therapeutic, and research challenges. The rarity of this combination severely limits the available evidence base, restricting research to case reports and small retrospective studies. These reports often exhibit heterogeneity in their methodology, clinical description, and reported outcomes, making it difficult to draw generalizable conclusions or establish a gold standard for management. Vaginal cancer is frequently detected incidentally during evaluations for POP or unrelated symptoms, leading to delayed diagnosis and potentially worse prognosis. It is evident that HPV mapping and biomarker confirmation (e.g., p16, p53) are critical for identifying suspected neoplastic changes, particularly given the prognostic significance of HPV status. The scarcity of data complicates the identification and evaluation of optimal treatment modalities for this dual pathology. Thorough post-treatment analysis, observation, and follow-up of patients, essential for detecting recurrence, also pose significant challenges due to the lack of standardized protocols and limited long-term data.

## 6. Conclusions

The systematic review highlights the rarity of vaginal cancer co-occurring with uterovaginal prolapse, underscoring significant clinical and research challenges. Analysis of the limited literature revealed that patients were slightly older (mean age approximately 74 years) than those with vaginal cancer alone, consistent with epidemiological data indicating a median age of 60–70 years for vaginal cancer. The symptoms were consistent across most cases. The critical need for lesion biopsy prior to treatment is paramount to confirm malignancy, determine the histological subtype, and guide the therapy. Biopsy enables differentiation of squamous cell carcinoma from rarer subtypes and assessment of prognostic biomarkers such as p16, p53, or HPV status and treatment planning.

Surgery with prolapse correction and, if deemed necessary, radiotherapy provide promising short-term outcomes results in patients with vaginal carcinoma and complete genital prolapse, reducing morbidity and improving quality of life.

Native tissue reconstruction is preferred over synthetic meshes in the context of an oncological condition, to minimize irritation-related risks, such as HPV-independent carcinogenesis or recurrence. Synthetic meshes, while effective for POP correction, are associated with complications like mesh erosion and chronic irritation, which may contribute to neoplastic transformation in HPV-negative tumors. Native tissue repair, using autologous fascia or ligaments, reduces these risks, though it has higher long-term POP recurrence rates. This preference is supported by evidence linking chronic irritation from foreign bodies (e.g., pessaries) to HPV-independent vaginal squamous cell carcinoma. Patient follow-up, including gynecological examinations and imaging studies, is mandatory, with the frequency tailored to individual patients. Follow-up typically involves gynecological examinations, complemented by imaging (e.g., MRI, PET-CT) to detect potential recurrence. HPV-negative tumors require more intensive monitoring due to a higher recurrence risk. The rarity of this dual pathology and the lack of standardized guidelines underscore the need for further research to establish optimal management protocols and improve long-term outcomes.

## Figures and Tables

**Table 1:** Case reports of the coexistence of uterine prolapse and vaginal cancer.

First-Line Treatment	FIGO Stage	First Author/Reference	Country	Year	Age, Years	POPQ	Symptoms	Histology	HPV Status	Further Treatment	Follow-Up
EBRT	Ib	Acharya [15]	Nepal	2012	84	III	vaginal discharge	squamous cell carcinoma	n/a	No	n/a
chemotherapy	IVb	Akino [10]	Japan	2016	60	III	vaginal bleeding	squamous cell carcinoma	HPV (−) negative	No	2 months, DoD
RH, partial colpectomy, PLND, omentectomy	I	Babarović [16]	Croatia	2017	82	III	vaginal bleeding	carcinosarcoma + squamous intraepithelial neoplasia	P16 (−) P53(+)	No	14 months, NED
partial colpectomy	I	Batista [17]	Brazil	2008	73	III	vaginal bleeding	squamous cell carcinoma	n/a	EBRT	24 months, NED
VH, partial colpectomy, levator myorrhaphy	IIb	Buda [18]	Italy	2020	81	IV	vaginal discharge and bleeding	squamous cell carcinoma	n/a	BT	10 months, NED
partial colpectomy +GLND	n/a	Dellino [19]	Italy	2020	82	III	vaginal bleeding	squamous cell carcinoma	n/a	EBRT+BT	24 months, DoD with preexisting cardiopathy
palliation (nephrostomy)	IVa	Fedus [20]	Poland	2017	69	IV	bulging and urinary incontinence	squamous cell carcinoma	n/a	No	n/a
RH, excision of the perforation of the vaginal site and adnexa, necrotic intestine resection	n/a	Fukai [21]	Japan	2023	90	IV	abdominal pain, protruding mass	squamous cell carcinoma	p16(−), p53(+), HPV (−) negative	No	8 days, DoD
RVH with bilateral PLND with partial colpectomy	I	Ghosh [22]	India	2008	50	III	bulging and voiding difficulties	squamous cell carcinoma	n/a	No	12 months, NED
pessary + EBRT with concomitant cisplatin + BT	II	Gultekin [23]	Turkey	2020	77	IV	vaginal bleeding	squamous cell carcinoma	n/a	No	10 months, NED
chemotherapy, 5-fluorouracil, carboplatin	III	Gupta [24]	India	2007	60	III	vaginal bleeding	squamous cell carcinoma	n/a	No	n/a
RVH with partial EBRT colpectomy	I	Iavazzo [25]	Greece	2007	80	III	vaginal bleeding	squamous cell carcinoma	n/a	EBRT	42 months, NED
BT	I	Ishibashi [26]	Japan	2019	78	IV	vaginal bleeding	squamous cell carcinoma	n/a	No	3 months, NED
none	n/a	Jain [13]	United Kingdom	2009	88,96	n/a	vaginal bleeding	sarcomatoid squamous cell carcinoma and squamous cell carcinoma	n/a	No	Both few months, DoD and DoOR
SH, cervicopexy to pectineal ligaments by polypropylene mesh	II	Karateke [27]	Turkey	2006	68	III	bulging	squamous cell carcinoma	n/a	EBRT	20 months, NED
none (palliative chemotherapy was planned)	IV	Kim [28]	South Korea	2014	80	IV	bulging, vaginal bleeding, pelvic pain	squamous cell carcinoma	n/a	No	1 month, DoD
RH, PLND	I	Korniluk	Poland	2024	77	IV	bulging	squamous cell carcinoma (keratizing)	HPV (−) negative, P16 unspecific, P53+	EBRT+BT	12 months, NED
EBRT + cisplatin	n/a	Kowalski [29]	USA	2015	82	IV	urinary incontinence, worsening vaginal pressure, incomplete bladder emptying	squamous cell carcinoma	n/a	No	4 months, cause of death unknown
RH, PLND, left partial cystectomy with ureteral reimplantation and total colpectomy	IVa	Malek [30]	Tunisia	2020	77	III	bulging	warty squamous cell carcinoma	n/a	EBRT	93 months, DoOR
VH partial colpectomy, suspension to round ligaments	n/a	Moszyński [31]	Poland	2014	65	IV	n/a	squamous cell carcinoma	n/a	polypropylene colposacropexy for early vault prolapse recurrence + BT	36 months, NED
five EBRT, one refused treatment	n/a	Rao [32]	India	1989	44–72	III	n/a	squamous cell carcinoma	n/a	No	n/a
referred to EBRT (but lost to follow-up)	I	Singhal [33]	India	2017	62	II	vaginal bleeding	squamous cell carcinoma	n/a	No	n/a
VH	n/a	Sheikh [34]	India	2019	72	III	bulging	squamous cell carcinoma	n/a	EBRT	n/a
1. VH, BSO, sacrospinous fixation. 2. neoadjuvant chemotherapy. 3. refused treatment	n/a	Tan [12]	Malaysia	2021	72–98	III-IV	bulging, vaginal bleeding	squamous cell carcinoma	n/a	EBRT	1. few months, DoD 2. n/a, DoOR 3. 12 months, DoD
VH, radical colpo-vulvectomy, GLND, cystocele and rectocele repair	I	Vijay Kumar [35]	India	2013	80	III	bulging	squamous cell carcinoma	n/a	No	6 months, NED
VH, partial colpectomy, apex fixation, anterior and posterior colporrhaphy	I	Wang [36]	China	2014	61	III	bulging	squamous cell carcinoma	n/a	EBRT	48 months, NED

Abbreviations: **POPQ** pelvic organ prolapse qualification, **FIGO** International Federation of Gynecology and Obstetrics, **HPV** human papilloma virus, **n/a** not available, **EBRT** external beam radiotherapy, **NED** no evidence of disease, **BT** brachytherapy, **RH** radical hysterectomy, **PLND** pelvic lymphadenectomy, **VH** vaginal hysterectomy, **GLND** groin lymphadenectomy, **RVH** radical vaginal hysterectomy, **SH** subtotal hysterectomy, **BSO** bilateral salpingo-oophorectomy, **DoD** dead of disease, **DoOR** dead of other reason.

## Data Availability

The original contributions presented in this study are included in the article/supplementary Material. Further inquiries can be directed to the corresponding author.

## Supplementary Materials

The following supporting information can be downloaded at https://www.mdpi.com/article/10.3390/jcm14134622/s1.
