# Supplementary material for: Primary Invasive Vaginal Carcinoma Associated with Complete Utero-Vaginal Prolapse: A Case Report and Literature Review
Source: J Clin Med. 2025 Jun 30;14(13):4622. doi: 10.3390/jcm14134622 (PMC12250043; doi:10.3390/jcm14134622)
Supplement: Supplementary file 1 [file jcm-14-04622-s001.zip › Supplementary Material S1..pdf]

Supplementary Table 1. Search strategy. (Pubmed)

	**Pelvic organ prolapse AND vaginal cancer 217**
#1	(("pelvic organ prolapse"[MeSH Terms] OR ("pelvic"[All Fields] AND "organ"[All Fields] AND "prolapse"[All Fields]) OR "pelvic organ prolapse"[All Fields]) AND ("vaginal neoplasms"[MeSH Terms] OR ("vaginal"[All Fields] AND "neoplasms"[All Fields]) OR "vaginal neoplasms"[All Fields] OR ("vaginal"[All Fields] AND "cancer"[All Fields]) OR "vaginal cancer"[All Fields])) AND (1945:2024[pdat])
	**Pelvic organ prolapse AND vaginal carcinoma 39**
#2	(("pelvic organ prolapse"[MeSH Terms] OR ("pelvic"[All Fields] AND "organ"[All Fields] AND "prolapse"[All Fields]) OR "pelvic organ prolapse"[All Fields]) AND ("vagina"[MeSH Terms] OR "vagina"[All Fields] OR "vaginal"[All Fields] OR "vaginally"[All Fields] OR "vaginals"[All Fields] OR "vaginitis"[MeSH Terms] OR "vaginitis"[All Fields] OR "vaginitides"[All Fields]) AND ("carcinoma"[MeSH Terms] OR "carcinoma"[All Fields] OR "carcinomas"[All Fields] OR "carcinoma s"[All Fields])) AND (1948:2024[pdat])
	**Pelvic organ prolapse AND vaginal neoplasm 124**
#3	(("pelvic organ prolapse"[MeSH Terms] OR ("pelvic"[All Fields] AND "organ"[All Fields] AND "prolapse"[All Fields]) OR "pelvic organ prolapse"[All Fields]) AND ("vaginal neoplasms"[MeSH Terms] OR ("vaginal"[All Fields] AND "neoplasms"[All Fields]) OR "vaginal neoplasms"[All Fields] OR ("vaginal"[All Fields] AND "neoplasm"[All Fields]) OR "vaginal neoplasm"[All Fields])) AND (1948:2024[pdat])
	**Genital prolapse AND vaginal carcinoma 87**
#4	(("genitalia"[MeSH Terms] OR "genitalia"[All Fields] OR "genital"[All Fields] OR "genitals"[All Fields] OR "genitally"[All Fields]) AND ("uterine prolapse"[MeSH Terms] OR ("uterine"[All Fields] AND "prolapse"[All Fields]) OR "uterine prolapse"[All Fields] OR ("prolapse"[All Fields] AND "vaginal"[All Fields]) OR "prolapse vaginal"[All Fields]) AND ("carcinoma"[MeSH Terms] OR "carcinoma"[All Fields] OR "carcinomas"[All Fields] OR "carcinoma s"[All Fields])) AND (1948:2024[pdat])
	**Genital prolapse AND vaginal cancer 316**

#5	(("genitalia"[MeSH Terms] OR "genitalia"[All Fields] OR "genital"[All Fields] OR "genitals"[All Fields] OR "genitally"[All Fields]) AND ("uterine prolapse"[MeSH Terms] OR ("uterine"[All Fields] AND "prolapse"[All Fields]) OR "uterine prolapse"[All Fields] OR ("prolapse"[All Fields] AND "vaginal"[All Fields]) OR "prolapse vaginal"[All Fields]) AND ("cancer s"[All Fields] OR "cancerated"[All Fields] OR "canceration"[All Fields] OR "cancerization"[All Fields] OR "cancerized"[All Fields] OR "cancerous"[All Fields] OR "neoplasms"[MeSH Terms] OR "neoplasms"[All Fields] OR "cancer"[All Fields] OR "cancers"[All Fields])) AND (1945:2024[pdat])
	**Genital prolapse AND vaginal neoplasm 258**
#6	(("genitalia"[MeSH Terms] OR "genitalia"[All Fields] OR "genital"[All Fields] OR "genitals"[All Fields] OR "genitally"[All Fields]) AND ("uterine prolapse"[MeSH Terms] OR ("uterine"[All Fields] AND "prolapse"[All Fields]) OR "uterine prolapse"[All Fields] OR ("prolapse"[All Fields] AND "vaginal"[All Fields]) OR "prolapse vaginal"[All Fields]) AND ("neoplasm s"[All Fields] OR "neoplasms"[MeSH Terms] OR "neoplasms"[All Fields] OR "neoplasm"[All Fields])) AND (1948:2024[pdat])
	**Uterus prolapse AND vaginal carcinoma 51**
#7	(("uterine prolapse"[MeSH Terms] OR ("uterine"[All Fields] AND "prolapse"[All Fields]) OR "uterine prolapse"[All Fields] OR ("uterus"[All Fields] AND "prolapse"[All Fields]) OR "uterus prolapse"[All Fields]) AND ("vagina"[MeSH Terms] OR "vagina"[All Fields] OR "vaginal"[All Fields] OR "vaginally"[All Fields] OR "vaginals"[All Fields] OR "vaginitis"[MeSH Terms] OR "vaginitis"[All Fields] OR "vaginitides"[All Fields]) AND ("carcinoma"[MeSH Terms] OR "carcinoma"[All Fields] OR "carcinomas"[All Fields] OR "carcinoma s"[All Fields])) AND (1948:2024[pdat])
	**Uterus prolapse AND vaginal cancer 159**
#8	(("uterine prolapse"[MeSH Terms] OR ("uterine"[All Fields] AND "prolapse"[All Fields]) OR "uterine prolapse"[All Fields] OR ("uterus"[All Fields] AND "prolapse"[All Fields]) OR "uterus prolapse"[All Fields]) AND ("vaginal neoplasms"[MeSH Terms] OR ("vaginal"[All Fields]

	AND "neoplasms"[All Fields]) OR "vaginal neoplasms"[All Fields] OR ("vaginal"[All Fields] AND "cancer"[All Fields]) OR "vaginal cancer"[All Fields])) AND (1945:2024[pdat])
	**Uterus prolapse AND vaginal neoplasm 137**
#9	(("uterine prolapse"[MeSH Terms] OR ("uterine"[All Fields] AND "prolapse"[All Fields]) OR "uterine prolapse"[All Fields] OR ("uterus"[All Fields] AND "prolapse"[All Fields]) OR "uterus prolapse"[All Fields]) AND ("vaginal neoplasms"[MeSH Terms] OR ("vaginal"[All Fields] AND "neoplasms"[All Fields]) OR "vaginal neoplasms"[All Fields] OR ("vaginal"[All Fields] AND "neoplasm"[All Fields]) OR "vaginal neoplasm"[All Fields])) AND (1948:2024[pdat])
	**Uterine prolapse AND vaginal carcinoma 129**
#10	(("uterine prolapse"[MeSH Terms] OR ("uterine"[All Fields] AND "prolapse"[All Fields]) OR "uterine prolapse"[All Fields]) AND ("vagina"[MeSH Terms] OR "vagina"[All Fields] OR "vaginal"[All Fields] OR "vaginally"[All Fields] OR "vaginals"[All Fields] OR "vaginitis"[MeSH Terms] OR "vaginitis"[All Fields] OR "vaginitides"[All Fields]) AND ("carcinoma"[MeSH Terms] OR "carcinoma"[All Fields] OR "carcinomas"[All Fields] OR "carcinoma s"[All Fields])) AND (1948:2024[pdat])
	**Uterine prolapse AND vaginal cancer 461**
#11	(("uterine prolapse"[MeSH Terms] OR ("uterine"[All Fields] AND "prolapse"[All Fields]) OR "uterine prolapse"[All Fields]) AND ("vaginal neoplasms"[MeSH Terms] OR ("vaginal"[All Fields] AND "neoplasms"[All Fields]) OR "vaginal neoplasms"[All Fields] OR ("vaginal"[All Fields] AND "cancer"[All Fields]) OR "vaginal cancer"[All Fields])) AND (1945:2024[pdat])
