# Supplementary material for: Primary Invasive Vaginal Carcinoma Associated with Complete Utero-Vaginal Prolapse: A Case Report and Literature Review
Source: J Clin Med. 2025 Jun 30;14(13):4622. doi: 10.3390/jcm14134622 (PMC12250043; doi:10.3390/jcm14134622)
Supplement: Supplementary file 1 [file jcm-14-04622-s001.zip › Supplementary Material S2..pdf]

Quality assessment- The CARE guidelines comprise thirteen items grouped into key domains, such as patient information, clinical narrative, therapeutic interventions, and discussion. A checklist approach is employed to evaluate the quality of a case report, assessing the lucidity of the case narrative, the suitability of diagnostic and treatment strategies, and the thoroughness of follow-up. Each item is marked as fulfilled or not fulfilled to ensure the report's transparency and comprehensiveness. High-quality case report satisfy all thirteen items, though some, such as the patient perspective, may be optional depending on the context, allowing for flexible evaluation tailored to the specifics of the case

Data extraction process- Data selection and extraction was conducted in accordance with PICOS using a piloted form specifically designed for capturing information on study and characteristics. Data were extracted independently by two authors to ensure accuracy and consistency.

Potential bias- The literature review may be subject to selection bias due to the inclusion of only full-text case reports, studies describing patients with vaginal cancer and pelvic organ prolapse, studies published between 1945 and 2024, and articles written in English, sourced exclusively from PubMed and manual searching of reference lists of key articles. These restrictions, driven by resource constraints and accessibility, may have excluded relevant non-full-text studies, cases with other conditions, earlier or unpublished research, non-English publications, or studies not indexed in PubMed or cited in the selected articles. Additionally, the review may be affected by publication bias, as unpublished studies or those with negative results were not included. To mitigate these biases, future reviews should incorporate additional databases (e.g., Scopus, Embase) and non-English publications
